# A qualitative study of pregnant women’s perceptions and decision-making regarding COVID-19 vaccination in Thailand

**DOI:** 10.1038/s41598-024-55867-z

**Published:** 2024-03-01

**Authors:** Saifon Chawanpaiboon, Sanitra Anuwutnavin, Attapol Kanjanapongporn, Julaporn Pooliam, Vitaya Titapant

**Affiliations:** 1 Division of Maternal-Fetal Medicine, Department of Obstetrics & Gynaecology, Faculty of Medicine Siriraj Hospital, Mahidol University, Bangkok, 10700 Thailand; 2 Department of Social Sciences, Faculty of Social Sciences and Humanities, Mahidol University, Nakhon Pathom, 73170 Thailand; 3 Clinical Epidemiological Unit, Office for Research and Development, Faculty of Medicine, Siriraj Hospital, Mahidol University, Bangkok, 10700 Thailand

**Keywords:** Acceptance, Attitude, COVID-19, Pregnant women, Rejection, Vaccination, Diseases, Health occupations, Medical research

## Abstract

To identify pregnant women’s attitudes towards, and acceptance and rejection of, COVID-19 vaccination. This prospective, descriptive, implementation study was conducted in the Antenatal clinic of Siriraj Hospital, Bangkok, Thailand. In Phase I, 40 pregnant women were interviewed. Phase II consisted of questionnaire development and data validation. In Phase III, the questionnaire was administered to 400 participants. Pregnant women’s attitudes towards and acceptance and rejection of COVID-19 vaccination. Most pregnant women were uncertain about the potential harm of vaccination to themselves or their unborn child, including risks such as miscarriage or premature birth (59–66/101 [58.4%–65.3%]; OR 2.53–8.33; 95% CI 1.23–3.60, 5.17–19.30; *P* < 0.001) compared to those who disagreed with vaccination. Their vaccination decisions were significantly influenced by social media information regarding vaccination complications in pregnant women (74/101 [73.3%]; OR 15.95; 95% CI 2.15–118.55; *P* = 0.001) compared to those who disagreed with vaccination. Most pregnant women opined that they should not receive a COVID-19 vaccination during pregnancy (adjusted odds ratio [AOR] 6.57; 95% CI 2.44–17.70; *P* = 0.001). Most also rejected vaccination despite being aware of its benefits (AOR 17.14; 95% CI 6.89–42.62; *P* < 0.001). Social media messages and obligatory vaccination certifications influence maternal vaccination decisions. Pregnant women believe vaccination helps prevent COVID-19 infection and reduces its severity. Nevertheless, the primary reason for their refusal was concern about potential harm to their unborn child or themselves during pregnancy.

**The Thai clinical trials registry:** TCTR20211126006.

## Introduction

Coronavirus disease 2019 (COVID-19) is a novel infectious disease that was first reported in Wuhan, China, on 31 December 2019. The symptoms were ranging from the common cold to more severe illnesses. The new virus was named ‘SARS-CoV-2’, and COVID-19 was declared a global pandemic in March 2020^1^.

Pregnant women face an elevated risk of severe infections and are more prone to experiencing serious complications from COVID-19 compared to non-pregnant women. These complications may involve ICU admission, invasive ventilation, ECMO (extracorporeal membrane oxygenation), and even death^2–4^ Physiological changes in the immune system and circulatory blood flow during pregnancy make pregnant women susceptible to respiratory infection from SARS-CoV-2^5^ Serious COVID-19 infection during pregnancy can result in disabilities and abnormalities of the foetus, intrauterine foetal death, and neonatal death during the postpartum period^6^.

The United States Centers for Disease Control and Prevention (CDC) reported that pregnant women are one of the groups at high risk of developing severe symptoms when they are infected with COVID-19. Women infected with COVID-19 are more likely to get sick from the infection than non-pregnant people. They may need to be admitted to an intensive care unit or use a ventilator or special breathing device. Severe illness from COVID-19 during pregnancy can lead to death. There is also a risk of complications affecting the pregnancy and the unborn child, such as miscarriage or death. Pregnant women infected with coronavirus are at increased risk of preeclampsia, miscarriage and other complications from pregnancy^7^. Respiratory complications may also prolong their hospital stay. Pregnant women with severe illnesses from coronavirus infection may also die. Therefore, raising awareness of the potential hazards of COVID-19 infection during pregnancy and counselling pregnant women about the safety of COVID-19 vaccines is critical^8^.

Risk factors for severe COVID-19 during pregnancy or postpartum include medical complications, age over 25, living/working in high COVID-19 areas, and close proximity to potentially infected individuals^9^. Pandemic restrictions limited prenatal care, leading to increased stillbirths and preterm births^10–12^. The WHO in the Western Pacific region advocates for vaccination and other measures to control COVID-19, with many countries endorsing at least one vaccine since 2022^13^.

The United States CDC currently monitors COVID-19 vaccinations during pregnancy and maternal and foetal complications during the first trimester to better understand the impact of vaccination on pregnancy and unborn children. Recent data show that administering an mRNA COVID-19 vaccine during pregnancy reduces the risk of serious illness from COVID-19 infection^14–16^. Vaccinations during pregnancy have also been found to produce antibodies that can help protect babies from COVID-19 because antibodies have been found in babies’ umbilical cord blood^17–19^. A recent small study found that 57% of babies at 6 months born to mothers vaccinated during pregnancy had antibodies to COVID-19. This proportion was substantially higher than that for babies born to mothers with a COVID-19 infection during pregnancy: only 8% of these babies had antibodies to COVID-19^20^.

Fell and colleagues reported that neonates born to vaccinated mothers did not have an increased need for admission to a neonatal intensive care unit and had better APGAR scores than neonates born to unvaccinated mothers^21^. In addition, vaccination was not associated with an increased risk of postpartum haemorrhage or chorioamnionitis. Pregnant women are a high-risk group recommended for vaccination to avoid complications from the disease, hospitalisation, intensive care unit admission and death^21^. Worldwide evidence shows that COVID-19 vaccinations during pregnancy are not associated with maternal or neonatal adverse outcomes and are effective in preventing infection in mothers and neonates during the first few months of life.

Numerous medical organisations recommend COVID-19 vaccination of pregnant women throughout their pregnancy^21^. Among them are the United States CDC, the American College of Obstetricians and Gynecologists, the Society for Maternal–Fetal Medicine, and the American Society for Reproductive Medicine.

WHO also works with affiliated governments through the multi-channel COVAX (Covid-19 Vaccines Global Access Facility) to facilitate access to vaccines and the earliest possible distribution of primary vaccinations. WHO recommended that the highest priority be given to distributing initial vaccination stocks to high-risk groups within each country. These were physicians, nurses, health workers, the elderly and people with significant health compromises. COVID-19 vaccines have become widely available since 2022^10^. This means that the provision of vaccinations can be expanded to other important groups, such as pregnant women, and the general population^10,22^.

However, many pregnant women have decided not to get vaccinated against COVID-19. They adopted this stance despite the publication of reports confirming the safety of vaccinating pregnant women in all trimesters of pregnancy^17,23^. We wanted in-depth information on pregnant women’s attitudes towards vaccination against COVID-19 infection and their reasons for accepting or refusing vaccination. The information gathered would facilitate planning for the care of pregnant women in the scenario of a new wave of outbreaks.

## Materials and methods

This was a prospective, descriptive study. The Siriraj Ethics Committee of the Faculty of Medicine at Siriraj Hospital approved the protocol (Si1018/2021). The Thai Clinical Trials registration number is TCTR20211126006.

This survey study utilised validated questionnaires (Phase III). The sample size was calculated using a proportion of the results of interest of 50% (*P* = 0.5), an estimation error of ≤ 5% and a 95% confidence level (type I error = 0.05; 2-sided). The number of pregnant women who needed to be surveyed was ≥ 385.

The research was divided into 3 consecutive phases: (1) in-depth interview; (2) questionnaire development and validation; (3) questionnaire administration (Supplementary Information).

### Phase I: in-depth interviews

To find participants for Phases I and III, pregnant women of any gestational age attending the hospital’s antenatal ward without any restriction were invited to a private counselling room where the research project was described. The pregnant women participating in the study were Thai women aged at least 16 years and literate. The women were given time to ask questions and consider whether they wished to enrol in the trial. They were advised that they could decline to participate or withdraw at any stage and for any reason if they did enrol. All women who volunteered to be research subjects for Phases I and III were asked to sign an informed consent form.

This phase collected information about the following 4 areas:

1. General pregnant women information
2. Pregnant women attitudes towards COVID-19 vaccination, knowledge of complications of COVID-19 infection during pregnancy, and reasons for accepting or rejecting COVID-19 vaccination
3. Decision-making about COVID-19 vaccination
4. Frustrations with deciding whether to be vaccinated

We conducted in-depth interviews with pregnant women to explore their knowledge, attitudes, acceptance, and refusal of vaccination. The insights gathered from these interviews were then utilized to develop a questionnaire.

Between 1st May and 15nd June 2022, in-depth interviews for Phase I were conducted with 40 women. Before these interviews commenced, the participants were asked for permission for the conversations and their structured interview to be audio-recorded. Forty-five pregnant women were invited to participate in the in-depth interview phase. Five pregnant women declined the interview because they were uncomfortable while answering the question and being audio-recorded. The subjects initially completed an attitude assessment questionnaire. It dealt with their attitudes, knowledge of the complications of a COVID-19 infection during pregnancy, and their reasons for accepting or rejecting COVID-19 vaccination. Several other aspects were then investigated in a structured interview. One aspect related to their decision-making for COVID-19 vaccination and any frustration they felt before deciding to be vaccinated.

The total time from the commencement of the questionnaire until the completion of the comprehensive interview was approximately 30 min. The data integrity of the research questions was later verified. The completeness of data provided by patients in the basic demographic questions regarding their history of vaccination, type of vaccination, and acceptance or rejection was reexamined to ensure data integrity.

### Phase II: questionnaire development and validation

Between 16th June and 31st July 2022, the data from the provisional questionnaire (non-revised and non-validated questionnaire) and the in-depth interviews administered during Phase I were analysed to determine the means and standard deviations. Doing so enabled the questionnaire and the interview questions to be refined. The validity and reliability of the revised questionnaire and interview questions were tested before their use in the next phase. The revised questionnaire and interview questions underwent testing for validity and reliability before their implementation in the subsequent phase. A specialized statistician assessed the questionnaire's validity, focusing on double-barreled, confusing, and leading questions. To evaluate the test–retest reliability, the same respondents completed the questionnaire again one month after the initial completion. Forty pregnant women were given a revised questionnaire to test its validity and accuracy.

### Phase III: questionnaire administration (final phase)

Between 1st August and 31st October 2022, the validated questionnaires which has been tested for validity and accuracy, were given to 430 pregnant women (different group from Phase I) in the hospital’s antenatal ward during the final phase. Out of 430 pregnant women approached for the study, 30 declined to participate, resulting in a total recruitment of 400 women in this specific timeframe. There was no dropout of pregnant women during response to questionnaire.

### Statistical analysis

Descriptive statistics were used to summarise demographic data. Categorical data are presented as numbers and percentages. Continuous data are presented as mean ± standard deviation or median and range. All analyses were performed using PASW Statistics for Windows (version 18.0; SPSS Inc., Chicago, IL, USA). Multivariate analysis was performed using the forward stepwise and multiple logistic regression methods.

Univariate and multivariate logistic regression analysis were used to identify statistically significant independent factors association. Factors having a p-value of less than 0.10 in the univariate analysis were imported to multivariate logistic regression to control the effect of possible confounding factors. Factors were chosen using the forward-stepwise likelihood ratio approach in the multiple logistic regression model, and the adjusted odds ratio (AOR) with its 95% CI at a p-value of ≤ 0.05 was used to determine statistically significant association.

### Institutional review board statement

The research was approved by the Ethics Committee of the Siriraj Institutional Review Board (COA [Si1018/2021]). All procedures performed in studies are not involving human participants.

### Informed consent statement

Written, informed consent was obtained from all participants.

## Results

The majority received a COVID-19 vaccination before pregnancy (341 cases [85.3%]), with approximately half receiving 2 vaccine doses (189 cases [47.3%]; Table 1). A fifth of the respondents (91 cases [22.8%]) had contracted COVID-19 before their pregnancy (Table 1).

**Table 1:** Demographic data of pregnant women responding to questionnaire.

Demographic data	N = 400
History of COVID-19 vaccination	
No	24 (6.0%)
Yes	
Before pregnancy	341 (85.3%)
During pregnancy	133 (33.3%)
Maternal age (years)	30.9 (6.0); 16–45
Gravida	
1	160 (40.0%)
2	135 (33.8%)
> 2	105 (26.2%)
Miscarriage	
0	296 (74.0%)
≥ 1	104 (26.0%)
Pregnancy type	
Spontaneous	387 (96.8%)
Assisted reproductive technique (ICSI, IVF)	13 (3.3%)
Religion	
Buddhist	382 (95.5%)
Islam	15 (3.8%)
Christian	3 (0.8%)
Marital status	
Married	376 (94.0%)
Separated	8 (2.0%)
Other (single)	16 (4.0%)
Highest education level	
Below bachelor’s degree	217 (54.3%)
Bachelor’s degree or higher	183 (45.7%)
Family income (Baht/month)	
< 15,000	124 (31.0%)
15,001–30,000	183 (45.8%)
> 30,000	93 (23.2%)
Reimbursement of medical expenses	
Direct disbursement	102 (25.5%)
Social security	182 (45.5%)
Health insurance	52 (13.0%)
Other (self-pay, private insurance)	64 (16.0%)
History of other vaccinations during pregnancy	
Influenza	154 (38.5%)
Diphtheria, tetanus, pertussis	107 (26.8%)
Other	2 (0.5%)
Infected with COVID-19 before pregnancy	91 (22.8%)

**Figure 1:**
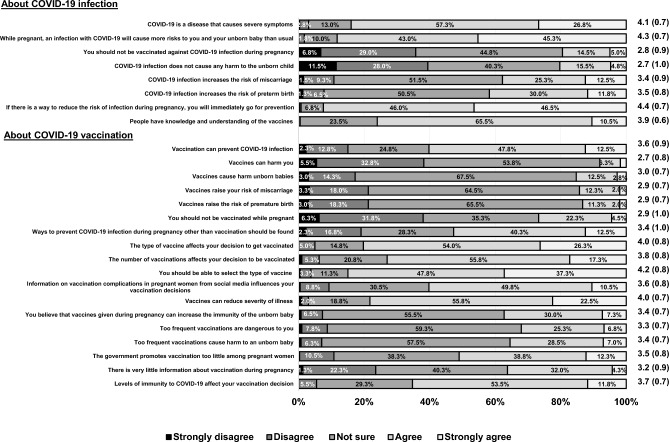
patient knowledge of complications of COVID-19 infection and COVID-19 vaccination during pregnancy.

More than 50% of the respondents agreed that COVID-19 is a potentially dangerous disease and can cause miscarriages and premature births. However, over 50% were unsure whether the vaccine could protect against COVID-19, might harm an unborn baby, or could increase the risk of miscarriage or preterm birth (Fig. 1). More than half (55.8%) were confident that the COVID-19 vaccine could reduce the severity of the disease.

**Figure 2:**
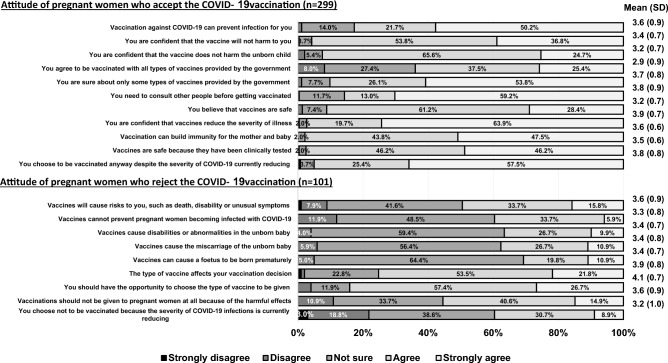
Patient attitude and acceptance and rejection of COVID-19 vaccination.

Of those who accepted the COVID-19 vaccination, more than 50% believed that the vaccine would protect against COVID-19, 65.6% were unsure whether the vaccine would harm their child, and 59.2% were still unsure and needed to consult others before getting vaccinated. Of those who refused to be vaccinated against COVID-19, more than 50% were unsure if the vaccine could cause foetal abnormalities or increase the risk of miscarriage or preterm birth (Fig. 2).

More than 70% of the pregnant women indicated that the core information that influenced their vaccination decisions was the following:

1. the severity of COVID-19 illness
2. the reduction in infection and disease severity resulting from vaccination
3. the potential harm to an unborn baby caused by COVID-19 vaccines
4. the level of immunity to COVID-19 in women who have been previously vaccinated (Table 2)

**Table 2:** Information for making decisions about COVID-19 vaccination (can select more than one choice).

Relevant information	N = 400
Your opinion of the seriousness of COVID-19 infection	
Very serious	290 (72.5%)
Moderate	57 (14.2%)
Mild	11 (2.8%)
Unsure	42 (10.5%)
COVID-19 vaccination can prevent infections and reduce the severity of symptoms	
Know	336 (84.0%)
Don’t know	14 (3.5%)
Unsure	50 (12.5%)
Obtained doctor or healthcare professional advice about vaccination	
Yes	323 (80.8%)
No	77 (19.3%)
Doctor or healthcare professional advised you that vaccination can reduce risk of infection	
Yes	370 (92.5%)
No	30 (7.5%)
You know that vaccinations can be performed during pregnancy	
Yes	338 (84.5%)
No	60 (15.0%)
Unsure	2 (0.5%)
If vaccination is helpful, you will	
Get an injection	238 (59.5%)
Need to consult relatives	116 (29.0%)
Reject injection	46 (11.5%)
The people who influence your decision are	
Husband	253 (63.2%)
Parents	157 (39.3%)
Sibling	30 (7.5%)
Other (eg, another relative; your doctor)	45 (11.3%)
Reason for rejecting vaccination	
Fear of pain	2 (0.5%)
Fear of harm to yourself	48 (12.0%)
Fear of harm to the unborn child	321 (80.3%)
Wanted to choose an alternative vaccine to the type allocated by the government	59 (14.8%)
Worried that if not vaccinated, there may be problems with healthcare workers	3 (0.8%)
Other	13 (3.3%)
If alternative vaccinations cost not much more, would you decide to be vaccinated?	
Would be vaccinated	246 (61.5%)
Would not be vaccinated	132 (33.0%)
Other	22 (5.5%)
The severity of a COVID-19 infection made you decide not to be vaccinated	
Yes	69 (17.3%)
No	240 (60.0%)
Unsure	91 (22.8%)
Your opinion on the types of vaccines that have been allocated by the government	
You should have the opportunity to choose your own type of vaccine	215 (53.8%)
Your doctor should advise you on the type of vaccine	115 (28.7%)
The government must fund vaccination costs throughout the epidemic	95 (23.8%)
Ready and willing to pay for alternative vaccines	19 (4.8%)
Other	4 (1.0%)
Your opinion on the vaccination process according to the policy of the Ministry of Public Health	
Lack of good publicity from the Ministry of Health on vaccinating pregnant women	123 (30.8%)
Doctors and healthcare professionals do not provide information and advice on vaccinating pregnant women	70 (17.5%)
Immunity levels for COVID-19 should be provided before getting a vaccination	234 (58.5%)
Other	13 (3.3%)
Ways to prevent COVID-19 infection that are better than vaccination	
Washing of your hands	208 (52.0%)
Alcohol gel use	170 (42.5%)
Social distancing	293 (73.3%)
Other	21 (5.3%)
If you have been vaccinated before pregnancy, you are confident that there will be no need for further vaccinations when you are pregnant	
Yes	112 (28.0%)
No	152 (38.0%)
Unsure	136 (34.0%)
If you have been vaccinated before pregnancy, you would like to know your immunity level before deciding to get vaccinated during pregnancy	
Yes	283 (70.8%)
No	49 (12.3%)
Unsure	68 (17.0%)
If you have a low immunity level, you will decide to be vaccinated against COVID-19 during pregnancy	
Yes	218 (54.5%)
No	57 (14.2%)
Unsure	125 (31.3%)
Booking appointments for vaccinations is convenient	
Yes	229 (57.3%)
No	61 (15.3%)
Unsure	110 (27.5%)
It is difficult to book appointments for vaccinations	
Yes	97 (24.3%)
No	188 (47.0%)
Unsure	115 (28.7%)

More than 50% of the women reported that obligatory vaccination certifications impacted their daily work and their decisions to be vaccinated. Furthermore, over 50% indicated that social media information about the dangers of vaccination or the death of pregnant women influenced their vaccination decisions. Additionally, more than 60% of the women agreed that the type, number of vaccinations, and levels of immunity to COVID-19 affected their vaccination decisions (Table 3).

**Table 3:** Grievances about the decision to get a vaccination.

Relevant information	N = 400
You felt embarrassed when your doctor advised you to get a vaccination	
Yes	73 (18.3%)
No	273 (68.3%)
Unsure	54 (13.5%)
You believed that your doctor would not provide standardised ante-natal care if you did not get vaccinated as recommended by the doctor	
Yes	66 (16.5%)
No	254 (63.5%)
Unsure	80 (20.0%)
People are forced by doctors to get vaccinated	
Yes	5 (1.3%)
No	379 (94.8%)
Unsure	16 (4.0%)
People are forced by their husbands or close relatives to get vaccinated	
Yes	5 (1.3%)
No	869 (96.5%)
Unsure	9 (2.3%)
You are forced by your employer to get vaccinated to obtain a certification of vaccination even if you do not want to be vaccinated	
Yes	28 (7.0%)
No	360 (90.0%)
Unsure	12 (3.0%)
Vaccination certification has a huge influence on your work and daily life	
Yes	234 (58.5%)
No	122 (30.5%)
Unsure	44 (11.0%)
Social media information and stories about the dangers to, and deaths of, pregnant women influence your vaccination decision	
Yes	221 (55.3%)
No	103 (25.8%)
Unsure	76 (19.0%)
The type of vaccine influences your vaccination decision	
Yes	289 (72.3%)
No	72 (18.0%)
Unsure	39 (9.8%)
The number of previous injections influences your next vaccination decision	
Yes	246 (61.5%)
No	102 (25.5%)
Unsure	52 (13.0%)
Information from the government or the Ministry of Health influences your vaccination decision	
Yes	266 (66.5%)
No	86 (21.5%)
Unsure	48 (12.0%)
Your COVID-19 immunity level influences your vaccination decision	
Yes	283 (70.8%)
No	71 (17.8%)
Unsure	46 (11.5%)

Of the 101 pregnant women who rejected having a COVID-19 vaccination, most were unsure whether the vaccine could harm themselves (59/101 [58.4%]; odds ratio [OR] 8.26; 95% CI 3.59–19.01; *P* < 0.001) or their unborn child (66/101 [65.3%]; OR 5.26; 95% CI 1.85–14.98; *P* < 0.001), including miscarriage (61/101 [60.4%]; OR 8.33; 95% CI 3.60–19.30; *P* < 0.001) or premature birth (66/101 [65.3%]; OR 2.53; 95% CI 1.23–5.17; *P* < 0.001; Table 4). They believed that too many vaccinations might harm themselves (43/101 [42.6%]; OR 3.04; 95% CI 1.10–8.38; *P* = 0.018) or their unborn child (48/101 [47.5%]; OR 4.26; 95% CI 1.22–14.81; *P* = 0.006; Table 4).

**Table 4:** Univariable analysis of factors associated with vaccination rejection (only factors that are likely to have a relationship of *P* < 0.10).

Factor	Reject (n = 101)	Accept (n = 299)	Odds ratio (95% CI)	*P* value
General information				
The highest level of education				
Below bachelor’s degree	62 (61.4%)	155 (51.8%)	1.45 (0.93–2.34)	0.096
Bachelor’s degree or higher	39 (38.6%)	144 (48.2%)	1.00	
Reimbursement of medical expenses				
Direct disbursement	29 (28.7%)	73 (24.4%)	1.67 (0.95–2.94)	0.050
Social security	35 (34.7%)	147 (49.2%)	1	
Health insurance	19 (18.8%)	33 (11.0%)	2.42 (1.23–4.75)	
Other (self-pay, private insurance)	18 (17.8%)	46 (15.4%)	1.64 (0.85–3.17)	
History of other vaccinations during pregnancy				
Influenza	46 (45.5%)	108 (36.1%)	1.48 (0.94–2.34)	0.092
Diphtheria, tetanus, pertussis	36 (35.6%)	71 (23.7%)	1.78 (1.09–2.89)	0.020
Other	1 (1.0%)	1 (0.3%)	2.98 (0.19–48.09)	0.442
Have been infected with COVID-19 before pregnancy	30 (29.7%)	61 (20.4%)	1.65 (0.99–2.75)	0.054
Attitudes about COVID-19 infection				
You should not be vaccinated against COVID-19 infection during pregnancy				
Disagree	12 (11.9%)	131 (43.8%)	1	< 0.001
Unsure	52 (51.5%)	127 (42.5%)	4.47 (2.28–8.77)	
Agree	37 (36.6%)	41 (13.7%)	9.85 (4.70–20.64)	
People have knowledge and understanding about the COVID-19 vaccine				
Disagree	1 (1.0%)	1 (0.3%)	1	0.054
Unsure	32 (31.7%)	62 (20.7%)	0.52 (0.03–8.53)	
Agree	68 (67.3%)	236 (78.9%)	0.29 (0.02–4.67)	
Attitudes about COVID-19 vaccination				
Vaccines can harm you				
Disagree	23 (22.8%)	130 (43.5%)	1	< 0.001
Unsure	59 (58.4%)	156 (52.2%)	2.14 (1.25–3.65)	
Agree	19 (18.8%)	13 (4.3%)	8.26 (3.59–19.01)	
Vaccines cause harm to unborn babies				
Disagree	4 (4.0%)	65 (21.7%)	1	< 0.001
Unsure	66 (65.3%)	204 (68.2%)	5.26 (1.85–14.98)	
Agree	31 (30.7%)	30 (10.0%)	16.79 (5.44–51.86)	
Vaccines raise your risk of miscarriage				
Disagree	10 (9.9%)	75 (25.1%)	1	< 0.001
Unsure	61 (60.4%)	197 (65.9%)	2.32 (1.13–4.77)	
Agree	30 (29.7%)	27 (9.0%)	8.33 (3.60–19.30)	
Vaccines raise risk of premature birth				
Disagree	10 (9.9%)	75 (25.1%)	1	< 0.001
Unsure	66 (65.3%)	196 (65.6%)	2.53 (1.23–5.17)	
Agree	25 (24.8%)	28 (9.4%)	6.70 (2.86–15.70)	
You should not be vaccinated while pregnant				
Disagree	11 (10.9%)	141 (47.2%)	1	< 0.001
Unsure	38 (37.6%)	103 (34.4%)	4.73 (2.31–9.69)	
Agree	52 (51.5%)	55 (18.4%)	12.12 (5.89–24.93)	
Information on vaccination complications in pregnant women from social media influence vaccination decisions				
Disagree	1 (1.0%)	36 (12.0%)	19.75 (1.28–74.51)	0.001
Unsure	26 (25.7%)	96 (32.1%)	15.95 (2.15–	
Agree	74 (73.3%)	167 (55.9%)	118.55)	
Vaccines can help reduce severity of illness				
Disagree	4 (4.0%)	8 (2.7%)	1	0.008
Unsure	29 (28.7%)	46 (15.4%)	1.26 (0.35–4.57)	
Agree	68 (67.3%)	245 (81.9%)	0.56 (0.16–1.90)	
Vaccines given during pregnancy can increase the immunity of the unborn baby				
Disagree	10 (9.9%)	19 (6.4%)	1	0.091
Unsure	62 (61.4%)	160 (53.5%)	0.74 (0.32–1.67)	
Agree	29 (28.7%)	120 (40.1%)	0.46 (0.19–1.09)	
Too frequent vaccinations will be dangerous to you				
Disagree	5 (5.0%)	30 (10.0%)	1	0.018
Unsure	53 (52.5%)	184 (61.5%)	1.73 (0.64–4.67)	
Agree	43 (42.6%)	85 (28.4%)	3.04 (1.10–8.38)	
Too frequent vaccinations will cause harm to the unborn baby				
Disagree	3 (3.0%)	25 (8.4%)	1	0.006
Unsure	50 (49.5%)	180 (60.2%)	2.32 (0.67–7.98)	
Agree	48 (47.5%)	94 (31.4%)	4.26 (1.22–14.81)	
The decision to vaccinate against COVID-19				
COVID-19 vaccinations can help prevent infections and severe symptoms				
Know	79 (78.2%)	257 (86.0%)	1	0.027
Don’t know	2 (2.0%)	12 (4.0%)	0.54 (0.12–2.47)	
Unsure	20 (19.8%)	30 (10.0%)	2.17 (1.17–4.03)	
Doctor or healthcare professional should explain to you that vaccination can reduce your risk of infection				
Should explain	87 (86.1%)	283 (94.6%)	1	0.005
Don’t have to explain	14 (13.9%)	16 (5.4%)	2.85 (1.34–6.07)	
If you know that vaccination is helpful, will you receive injections or not?				
Get a vaccination of course	30 (29.7%)	208 (69.6%)	1	< 0.001
Need to consult relatives first	38 (37.6%)	78 (26.1%)	3.38 (1.96–5.82)	
Don’t ask for a vaccination	33 (32.7%)	13 (4.3%)	17.60 (8.34–37.16)	
Reason for rejecting vaccination				
Fear of pain	1 (1.0%)	1 (0.3%)	2.98 (0.19–48.09)	0.419
Fear of harming yourself	19 (18.8%)	29 (9.7%)	2.16 (1.01–1.61)	0.015
Fear of harm to the unborn child	88 (87.1%)	233 (77.9%)	1.92 (1.01–3.65)	0.045
Wanted to choose an alternative vaccine to the type allocated by the government	6 (5.9%)	53 (17.7%)	0.29 (0.12–0.70)	0.004
Other	6 (5.9%)	7 (2.3%)	2.64 (0.86–8.03)	0.101
If alternative vaccinations cost not much more, would you decide to be vaccinated?				
Would be vaccinated	39 (38.6%)	207 (69.2%)	1	< 0.001
Would not be vaccinated	57 (56.4%)	75 (25.1%)	4.03 (2.48–6.56)	
Other	5 (5.0%)	17 (5.7%)	1.56 (0.54–4.48)	
You decided not to be vaccinated because the severity of COVID-19 is currently reducing				
Yes	30 (29.7%)	39 (13.0%)	1	< 0.001
No	43 (42.6%)	197 (65.9%)	0.28 (0.16–0.51)	
Unsure	28 (27.7%)	63 (21.1%)	0.58 (0.30–1.11)	
You would like to know your immunity level before deciding to get vaccinated during pregnancy				
Yes	62 (61.4%)	221 (73.9%)	1	0.055
No	17 (16.8%)	32 (10.7%)	1.89 (0.99–3.64)	
Unsure	22 (21.8%)	46 (15.4%)	1.71 (0.95–3.05)	
If you have low immunity level, you would decide to be vaccinated against COVID-19 during pregnancy				
Yes	34 (33.7%)	184 (61.5%)	1	< 0.001
No	26 (25.7%)	31 (10.4%)	4.54 (2.40–8.58)	
Unsure	41 (40.6%)	84 (28.1%)	2.64 (1.57–4.56)	
Booking appointments for vaccinations is convenient				
Yes	43 (42.6%)	186 (62.2%)	1	0.001
No	17 (16.8%)	44 (14.7%)	1.67 (0.87–3.20)	
Unsure	41 (40.6%)	69 (23.1%)	2.57 (1.55–4.28)	
It is difficult to book appointments for vaccinations				
Yes	18 (17.8%)	79 (26.4%)	1	0.008
No	42 (41.6%)	146 (48.8%)	1.26 (0.68–2.34)	
Unsure	41 (40.6%)	74 (24.7%)	2.43 (1.28–4.61)	
Grievances about decision to get vaccinated against COVID-19				
You felt embarrassed when your doctor advised you to get a vaccination				
Yes	35 (34.7%)	38 (12.7%)	1	< 0.001
No	51 (50.5%)	222 (74.2%)	0.25 (0.14–0.43)	
Unsure	15 (14.9%)	39 (13.0%)	0.42 (0.20–0.89)	
You believed that your doctor would not provide standardized ante-natal care if you did not get vaccinated as recommended by the doctor				
Yes	24 (23.8%)	42 (14.0%)	1	0.020
No	53 (52.5%)	201 (67.2%)	0.46 (0.26–0.83)	
Unsure	24 (23.8%)	56 (18.7%)	0.75 (0.38–1.50)	
The type of vaccine influences your vaccination decision				
Yes	64 (63.4%)	225 (75.3%)	1	0.046
No	22 (21.8%)	50 (16.7%)	1.55 (0.87–2.74)	
Unsure	15 (14.9%)	24 (8.0%)	2.20 (1.09–4.44)	

Most pregnant women believed they should not be vaccinated against COVID-19 during pregnancy (adjusted odds ratio [AOR] 6.57; 95% CI 2.44–17.70; *P* = 0.001; Table 5). They opined that COVID-19 vaccines increase the risk of premature birth (AOR 5.57; 95% CI 1.69–18.37; *P* = 0.011). Most rejected vaccination despite knowing its benefits (AOR 17.14; 95% CI 6.89–42.62; *P* < 0.001; Table 5).

**Table 5:** Multivariable analysis (using the forward stepwise method: multiple logistic regression of factors associated with rejected vaccination (only factors that are likely to have a relationship *P* < 0.10).

Factor	Crude odds ratio (95% CI)	*P* value	Adjusted odds ratio (95% CI)	*P* value
General information				
History of other vaccinations while pregnant (influenza, diphtheria, tetanus, whooping cough)	1.48 (0.94–2.34)	0.092	3.84 (1.92–7.67)	< 0.001
History of COVID-19 infection before pregnancy	1.65 (0.99–2.75)	0.054	1.94 (1.01–3.74)	0.049
Attitudes about COVID-19 infection				
Should not be vaccinated against COVID-19 during pregnancy				
Disagree	1	< 0.001	1	0.001
Unsure	4.47 (2.28–8.77)		4.67 (1.94–11.25)	
Agree	9.85 (4.70–20.64)		6.57 (2.44–17.70)	
Attitudes about vaccination against COVID-19				
Vaccines increase risk of premature birth				
Disagree	1	< 0.001	1	0.011
Unsure	2.53 (1.23–5.17)		1.97 (0.75–5.19)	
Agree	6.70 (2.86–15.70)		5.57 (1.69–18.37)	
Decision to vaccinate against COVID-19				
If you know that vaccination is helpful, will you receive injections or not?				
Get a vaccination of course	1	< 0.001	1	< 0.001
Need to consult relatives first	3.38 (1.96–5.82)		2.31 (1.20–4.43)	
Don’t ask for a vaccination	17.60 (8.34–37.16)		17.14 (6.89–42.62)	
You do not request the vaccination due to fear of harm to yourself	2.16 (1.01–1.61)	0.015	2.94 (1.26–6.88)	0.013
If you have been vaccinated before pregnancy, you would like to know your immunity level before deciding to get vaccinated during pregnancy				
Yes	1	0.055	1	0.031
No	1.89 (0.99–3.64)		2.95 (1.22–7.11)	
Unsure	1.71 (0.95–3.05)		0.74 (0.33–1.66)	
It is difficult to book appointments for vaccinations				
Yes	1	0.008	1	0.005
No	1.26 (0.68–2.34)		2.75 (1.19–6.34)	
Unsure	2.43 (1.28–4.61)		4.26 (1.77–10.26)	
Grievances about decision to get vaccinated against coronavirus				
You believed that your doctor would not provide standardized ante-natal care if you did not get vaccinated as recommended by the doctor				
Yes	1	0.020	1	0.030
No	0.46 (0.26–0.83)		0.48 (0.23–0.99)	
Unsure	0.75 (0.38–1.50)		0.29 (0.12–0.74)	

## Discussion

We engaged in in-depth interviews with pregnant women to delve into their knowledge, attitudes, acceptance, and refusal of vaccination. The valuable insights obtained from these interviews were instrumental in shaping the authentic data used to construct a questionnaire.

In our study, 85.3% of the pregnant women were vaccinated before becoming pregnant and understood the potential severity of COVID-19. Nevertheless, once pregnant, 50% of this subgroup had no confidence in the vaccination. They were concerned about the dangers of the vaccine to themselves and their unborn children, especially miscarriage and premature birth. This concern was evident despite their being aware that the vaccine can reduce the severity of the disease.

Our research also found that the pregnant women’s level of immunity to COVID-19 did not affect their vaccination decisions. The effectiveness of vaccines varies depending on the vaccine type and evolves over time within the pregnant population. The variations in immunity can be influenced by factors such as maternal age and underlying diseases, body mass index, and gestational age^24^. Nevertheless, pregnant women continue to express concerns about their immunity following previous injections and are hesitant to receive any further vaccinations during pregnancy.

Even if they knew their immunity level, they still decided not to get vaccinated because they were concerned about possible harm to the unborn baby, miscarriage or preterm delivery. This attitude must be adjusted during the COVID-19 pandemic. Several studies have shown that vaccination against COVID-19 before and during pregnancy is safe, effective and beneficial to both the mother and child. The benefits of getting a COVID-19 vaccination during pregnancy far outweigh any potential adverse consequences^23,25–28^.

No COVID-19 vaccine contains a live virus, so the vaccines do not cause COVID-19 infection in recipients, including pregnant women and their foetuses^23,25–28^. However, our investigation found that most respondents were uncertain whether the vaccine was safe for themselves and their unborn children. The women were unsure whether the vaccine would help prevent infection in their unborn babies. Most also believed that multiple vaccinations would harm their unborn children. This lack of information made it very challenging for them to decide whether to be vaccinated while pregnant.

Regarding the safety of mRNA COVID-19 vaccines (Moderna and Pfizer-BioNTech), no problems have been found for women vaccinated with them before or during pregnancy or for their unborn children^23,25–28^. Data from studies in the United States, Europe and Canada show that their use during pregnancy is not associated with an increased risk of complications, such as preterm birth, miscarriage and postpartum haemorrhage^21,26,29^. There is no increased risk of miscarriage in pregnant women administered an mRNA COVID-19 vaccine before or during early pregnancy (before 20 gestational weeks)^25,26,28,29^. A study from Chicago found that COVID-19 vaccination in pregnant women before and during the first trimester was not associated with a risk of congenital malformations^30^.

The administration of 2 primary doses of a COVID-19 mRNA vaccine to mothers during their pregnancy helped protect babies younger than 6 months from being hospitalised due to COVID-19 infection. In our investigation, the majority (84%) of infants hospitalised with an infection were born to women not vaccinated during pregnancy^31^.

Our research found that the type and number of vaccinations influenced vaccination decisions. In Thailand, the Pfizer-BioNTech and Moderna COVID-19 vaccines are more popular than the other COVID-19 vaccines available in the country, and these 2 vaccines have been reported to be safe in pregnant women^23^. However, some vaccination centres in Thailand only provide 1 type of vaccine. Consequently, people seeking vaccination may find that their preferred vaccine is unavailable. If an alternative vaccine can be provided by allowing pregnant women to select the vaccine themselves, it would likely increase the vaccination rate among pregnant women.

In addition, our research found that vaccination decisions are influenced by social media news about the dangers to mothers and unborn children, including death and disability. Most of the pregnant women in our study rejected vaccination because they were uncertain whether vaccination would increase their foetuses’ immunity. Recent research has revealed the role of social media in disseminating information and potentially influencing people’s attitudes towards vaccination. Studies have also shown the positive potential of social media in public health interventions and overcoming vaccination hesitancy among mothers^32–35^. Therefore, there should be thorough scrutiny of the various roles of social media in disseminating information to the public and influencing individual behaviour in the context of public health activities. This approach will give pregnant women a correct understanding of COVID-19 vaccines.

Vaccination certifications also play a key role in pregnant women’s vaccination decisions. Attending workplaces or meetings involving large groups of people puts individuals at risk of contracting COVID-19. Therefore, most public and private organisations require employees attending workplaces to be vaccinated to the levels recommended by the Thai Ministry of Health. Employers may also require certification of COVID-19 vaccination status. These restrictive policies pressurise pregnant women to get vaccinated even if they disagree with having a vaccination.

WHO has commented that COVID-19 is a health emergency that does not give governments many choices in quickly returning the situation to normal. Regarding calls for the widespread use of COVID-19 vaccination certificates, WHO recognises that introducing such certificates is risky and may result in harm. The general use of the certificates may cause deviations from their initial objectives: to ensure continuity of care and to provide proof of vaccination status. Legal or ethical considerations may be raised by further potential uses for vaccination certificates, for example, public health surveillance, pharmacovigilance, research, and exemptions to public health and social measures. WHO cites legal obligations to protect patient data and the need to respect human rights and fundamental freedoms. To this end, WHO has recommended that data protection measures be in place before adopting digital vaccination certificates. It has also stressed that vaccination certificates must not be considered a substitute for health surveillance^36^.

Our study presented both similar and different results from the previous study about attitudes, acceptance and rejection of COVID-19 vaccination among breastfeeding women^34^. Both pregnant and breastfeeding women believed that vaccines can reduce infection and disease severity. The women’s COVID-19 immunity levels did not affect their acceptance or rejection of vaccination and some mothers rejected vaccination because of concerns about possible harm to them or their newborns. The safety of COVID-19 vaccination to the unborn and newborn babies and mothers is the main concerning of both pregnant and breastfeeding women. However the different results of pregnant women from breastfeeding women were the effect of social media messages and vaccination certifications to their decision. Pregnant women had more concern about those issue than breastfeeding women. Most of pregnant women were still working and COVID-19 vaccination certifications were important to their works. While breastfeeding women have a right for stop working up to 90 days, therefore vaccination certification is not required.

To enhance COVID-19 vaccination rates during pregnancy, it is essential to address the significant decline in pregnant women's confidence in vaccination. Targeted strategies involve implementing comprehensive education and communication campaigns to dispel misinformation and underscore the safety and benefits of vaccination for both mothers and unborn children. Specific measures include developing focused educational initiatives, employing communication strategies to counter social media influence, improving information accessibility about vaccine types, establishing clear certification guidelines for safety, and tailoring messaging to address concerns about potential harm to unborn babies. These efforts aim to increase vaccination acceptance among pregnant women, contributing to improved maternal and fetal health outcomes.

Our study is affected by some limitation. The study design was prospective cross-sectional study which represented the real situation of COVID-19 outbreak during that time. The study is limited by the exclusive recruitment of the sample from Siriraj Hospital. Despite this, it's crucial to recognize that Siriraj Hospital, functioning as both a medical school and a referral center in Bangkok, draws patients from diverse regions of Thailand seeking advanced prenatal care. The selection of 400 pregnant women aimed to represent a varied demographic from different parts of the country. Although participants were not randomly chosen and were exclusively from Siriraj Hospital, the study intended to capture the extensive demographics and geographic diversity inherent in the hospital's patient population. Most of pregnant women (85.3%) had a history of COVID-19 vaccination which would affect the decision making for repeated vaccination. Their actual attitude may be affected by the severity of disease and availability of database of COVID-19 vaccination during pregnancy at that time.

The strength of our study is the less of “socially desirable” bias. In phase I, participant was in-depth interviewed in a close area by only single interviewer and in phase III, pregnant women response questionnaire in a closed place. The respondent can present the actual attitude, acceptance or rejection of COVID-19 vaccination.

## Conclusions

Although COVID-19 severity has decreased, it is essential to note that this factor does not significantly influence pregnant women's decisions to accept or refuse vaccination. The study findings indicate that pregnant women hold the belief that vaccines play a crucial role in preventing infection and mitigating the severity of the disease. Nevertheless, their main reason for refusing injections is concern about potential harm to their unborn children and themselves during pregnancy. Social media communications and obligatory vaccination certifications influence maternal vaccination decisions. Providing accurate information through social media will enable pregnant women to better understand the role of vaccination in reducing the severity of the disease and the complications for mothers and babies both during pregnancy and after childbirth.

### Supplementary Information

Supplementary Information.

## Data Availability

The data that support the findings of this study are available from Faculty of Medicine, Siriraj Hospital, Mahidol University but restrictions apply to the availability of these data, which were used under license for the current study, and so are not publicly available. Data are however available from the authors (Email address: saifon.cha@mahidol.ac.th) upon reasonable request and with permission of Faculty of Medicinle, Siriraj Hospital, Mahidol University.
