# Supplementary material for: A qualitative study of pregnant women’s perceptions and decision-making regarding COVID-19 vaccination in Thailand
Source: Sci Rep. 2024 Mar 1;14:5128. doi: 10.1038/s41598-024-55867-z (PMC10907718; doi:10.1038/s41598-024-55867-z)
Supplement: Supplementary file 1 — Supplementary Information. [file 41598_2024_55867_MOESM1_ESM.docx]

**Research Project: Attitudes, Acceptance and Rejection of COVID-19 Vaccination in Pregnant Women: an implementation research**

**Project participant number.................**

**Explanation:**

1. The questionnaire is under the research project “Attitudes, Acceptance and Rejection of COVID-19 vaccination in Pregnant”.

2. There are 4 parts of the questionnaire.

• Part 1: general information of the respondents.

• Part 2: a questionnaire about “attitudes on COVID-19 vaccination” “complications of COVID-19 infection during pregnancy and after childbirth" "acceptance" and "rejection"

• Part 3: a questionnaire on the decision to vaccinate against COVID-19.

• Part 4: a questionnaire to assess grievances about the decision to receive the COVID-19 vaccination.

Part 1 General information of the respondents

1. Age..................years
2. Religion

🞎 Buddhism 🞎 Islam

🞎 Christ 🞎 other (specified) ……………………….

1. marital status

🞎 couple 🞎 divorce

🞎 husband died 🞎 other (specified) ……………………….

1. the highest level of education

🞎 Elementary Education 🞎 Junior High School

🞎 Higher Secondary School 🞎 Diploma/High Vocational Certificate

🞎 Bachelor's degree 🞎 Master's degree/Ph.D.

1. Family income (bahts)

🞎 < 9,000 🞎 9,001-15,000

🞎 15,001-20,000 🞎 20,001-25,000

🞎 25,001-30,000 🞎 > 30,000

1. Medical treatment right

🞎 direct disbursement 🞎 social security

🞎 health insurance 🞎 other (specified) ……………………….

1. Pregnancy methods

🞎natural 🞎 assist reproduction

1. Have a history of vaccination against COVID-19 in the past

🞎 No 🞎 Yes, specified……………………….

1. Previous vaccination history while pregnant

🞎 No 🞎 Yes, specified……………………….

**Part 2: An attitude questionnaire about "Covid-19 Vaccination"**

**Explanation: Please read the following statement one by one by. After considering the questions clearly, then choose to answer the level of your attitude towards the question by ticking  in only one space at the end of the question.**

| **Statement** | **highly agree** | **agree** | **unsure** | **disagree** | **Highly disagree** |
| --- | --- | --- | --- | --- | --- |
|  | | | | | |
| **Attitudes about COVID-19 infection** | | | | | |
| 1. COVID-19 infection is very violent. |  |  |  |  |  |
| 2. If you have been infected with COVID-19 during pregnancy, it will cause more harm to you and your unborn baby than usual. |  |  |  |  |  |
| 3. Even if COVID-19 infection will be very dangerous but when pregnant, they should not be vaccinated. |  |  |  |  |  |
| 4. COVID-19 infection does not cause harm to the unborn baby. |  |  |  |  |  |
| 5. COVID-19 infection raises the risk of miscarriage or other complications. |  |  |  |  |  |
| 6. If there is a way to reduce the risk of infection during pregnancy, you will be protected immediately. |  |  |  |  |  |
| 7. You have knowledge about the COVID-19 vaccine. |  |  |  |  |  |
| **Attitudes about vaccination against COVID-19** | | | | | |
| 1. Vaccination against COVID-19 is dangerous to you and your unborn baby. |  |  |  |  |  |
| 2. Vaccination can help prevent infection |  |  |  |  |  |
| 3. Vaccinations should not be given to pregnant women. |  |  |  |  |  |
| 4. If your unborn baby is at risk of COVID-19 infection, you will find ways to prevent. |  |  |  |  |  |
| 5. The type of vaccine affects your decision  to get the injection. |  |  |  |  |  |
| 6. You should be involved in the choice or  type of vaccine injected. |  |  |  |  |  |
| 7. Knowledge about the COVID-19 vaccine |  |  |  |  |  |
| **Attitudes about accepting the COVID-19 vaccination** | | | | | |
| 1. COVID-19 vaccination is helpful because it can prevent infection to you. |  |  |  |  |  |
| 2. COVID-19 vaccination is helpful because it can prevent infection to the fetus. |  |  |  |  |  |
| 3. You can vaccinate any type of vaccination provided by the government. |  |  |  |  |  |
| 4. You need to consult others before vaccination. |  |  |  |  |  |
| 5. You believed that the vaccine was very safe. |  |  |  |  |  |
| 6. You are sure the vaccine can prevent disease. |  |  |  |  |  |
| 7. You are confident that the vaccine does not cause harm to you and fetus. |  |  |  |  |  |
| **Attitude about refusal to vaccinate against COVID-19** | | | | | |
| 1. Vaccine will cause you harm, such as death or disability. |  |  |  |  |  |
| 2. You think vaccines cannot prevent COVID-19 infection in pregnant women. |  |  |  |  |  |
| 3. you think that vaccine will cause abnormalities in the fetus, miscarriage or premature birth. |  |  |  |  |  |
| 3. The type of vaccine affects your vaccination decisions. |  |  |  |  |  |
| 4. You should have the opportunity to choose the type of vaccine to inject |  |  |  |  |  |
| 5. Vaccination in pregnant women should not be done at all because there is danger. |  |  |  |  |  |

**Part 3 Questionnaire about COVID-19 vaccination decisions**

**Explanation: Please read the following statement one by one. After considering the questions clearly, then choose the answer that best matches your opinion by ticking  or x in the  box in front of the selected answer. Please provide reasons or additional explanations for each question that has prepared the area to specify.**

1. The severity of Covid-19 infection is

 very violent  mild  not violent

1. COVID-19 vaccination can help prevent infection and severe symptoms of infection.

 known  unknown

1. Doctor or healthcare professional should explain to you whether vaccination can reduce your risk of infection

 should explain  don't have to explain

1. Vaccinations can be performed during pregnancy

 known  unknown

1. If you know vaccination is helpful you will be injected or not.

 get an injection of course

 need to consult relatives first

 Not have an injection

1. If you need to consult your relatives first , the people who influenced your decisions is.

 husband  parents  sibling  others people…………..

1. In the event that you do not request the injection, the reason is.

 fear of pain

 fear of harm to yourself

 fear of harm to the unborn child

 select an alternative vaccine that is not yet available in Thailand.

 others: specified……………………………….

1. You request the vaccination, the reason is:

 Worried that you might be in danger of vaccination

 Worried that the child might be in danger of vaccination

 Worried that if you are not injected, there may be problems with the medical staff.

 others: specified……………………………….

1. If alternative vaccinations cost little more, you will decide to inject or not.

 injected, because...........................

 not injected, because...........................

 others: specified……………………………….

1. What is your opinion on the types of vaccines that have been allocated by the government?

 Should give you the opportunity to choose your own injection.

 The doctor should give advice on the injection guidelines and the type of vaccine injected because you have no knowledge.

 I want the government to take care and support for vaccination costs forever during epidemic periods

 Ready and willing to pay for alternative vaccines

 Other................................................................ .............

1. What is your opinion on the vaccination process according to the policy of the Ministry of Public Health?

 Well done is .................................................. ...

 still not doing well, specified....................................

 Other .................................................
